# Vitamin C Administration by Intravenous Infusion Increases Tumor Ascorbate Content in Patients With Colon Cancer: A Clinical Intervention Study

**DOI:** 10.3389/fonc.2020.600715

**Published:** 2021-01-11

**Authors:** Gabi U. Dachs, Jamish Gandhi, Christina Wohlrab, Anitra C. Carr, Helen R. Morrin, Juliet M. Pullar, Simone B. Bayer, Tim W. Eglinton, Bridget A. Robinson, Margreet C. M. Vissers

**Affiliations:** ^1^ Mackenzie Cancer Research Group, Department of Pathology and Biomedical Science, University of Otago Christchurch, Christchurch, New Zealand; ^2^ Department of Surgery, Christchurch Hospital, University of Otago Christchurch, Christchurch, New Zealand; ^3^ Nutrition in Medicine Research Group, Department of Pathology and Biomedical Science, University of Otago Christchurch, Christchurch, New Zealand; ^4^ Cancer Society Tissue Bank, University of Otago Christchurch, Christchurch, New Zealand; ^5^ Centre for Free Radical Research, Department of Pathology and Biomedical Science, University of Otago Christchurch, Christchurch, New Zealand; ^6^ Canterbury Regional Cancer and Haematology Service, Canterbury District Health Board, Department of Medicine, University of Otago Christchurch, Christchurch, New Zealand

**Keywords:** vitamin C, plasma, tumor, colorectal cancer, normal mucosa, tumor hypoxia, hypoxia-inducible factor

## Abstract

The use of high dose ascorbate infusions in cancer patients is widespread, but without evidence of efficacy. Several mechanisms whereby ascorbate could affect tumor progression have been proposed, including: (i) the localized generation of cytotoxic quantities of H_2_O_2_; (ii) ascorbate-dependent activation of the 2-oxoglutarate-dependent dioxygenases that control the hypoxia-inducible factors (HIFs) and that are responsible for the demethylation of DNA and histones; (iii) increased oxidative stress induced by dehydroascorbic acid. We hypothesize that the dysfunctional vasculature of solid tumors results in compromised delivery of ascorbate to poorly perfused regions of the tumor and that this ascorbate deficit acts as an additional driver of the hypoxic response via upregulation of HIFs. Using a randomized “therapeutic window of opportunity” clinical study design we aimed to determine whether ascorbate infusions affected tumor ascorbate content and tumor biology. Patients with colon cancer were randomized to receive infusions of up to 1 g/kg ascorbate for 4 days before surgical resection (*n* = 9) or to not receive infusions (*n* = 6). Ascorbate was measured in plasma, erythrocytes, tumor and histologically normal mucosa at diagnostic colonoscopy and at surgery. Protein markers of tumor hypoxia or DNA damage were monitored in resected tissue. Plasma ascorbate reached millimolar levels following infusion and returned to micromolar levels over 24 h. Pre-infusion plasma ascorbate increased from 38 ± 10 µM to 241 ± 33 µM (p < 0.0001) over 4 days and erythrocyte ascorbate from 18 ± 20 µM to 2509 ± 1016 µM (p < 0.005). Tumor ascorbate increased from 15 ± 6 to 28 ± 6 mg/100 g tissue (p < 0.0001) and normal tissue from 14 ± 6 to 21 ± 4 mg/100 g (p < 0.001). A gradient of lower ascorbate was evident towards the tumor centre in both control and infusion samples. Lower expression of hypoxia-associated proteins was seen in post-infusion tumors compared with controls. There were no significant adverse events and quality of life was unaffected by ascorbate infusion. This is the first clinical study to demonstrate that tumor ascorbate levels increase following infusion, even in regions of poor diffusion, and that this could modify tumor biology.

**Clinical Trial Registration:**

ANZCTR Trial ID ACTRN12615001277538 (https://www.anzctr.org.au/).

## Introduction

The use of high dose vitamin C (ascorbate) infusions as a complementary therapy for cancer is widespread (1), despite a lack of robust evidence for efficacy. Ascorbate is administered to cancer patients in amounts far in excess of the recommended daily intake, in the absence of any guidelines on recommended dosages. This practice was initiated when early clinical experiments in late stage cancer patients reported improved patient survival following daily intravenous infusion of around 10 g of ascorbate, compared to historical controls (2). However, randomized, placebo-controlled trials (RCT) from the Mayo Clinic failed to show significant benefit with the same amount of ascorbate given orally (3). Differences between the studies with respect to study design and methods of ascorbate administration are now thought to be significant in explaining this discrepancy.

Pharmacokinetic and modeling data have demonstrated that tight regulation of ascorbate uptake and excretion means that plasma concentrations do not readily exceed 100 µM following oral intake (4, 5). In contrast, intravenous ascorbate administration bypasses uptake regulation in the gastrointestinal tract and results in dose-dependent increases in plasma levels, even in excess of 20 mM (4, 6–8). These levels are not maintained following infusion and ascorbate is cleared with a half-life of < 2 h (4, 6–8). That this difference in pharmacokinetics may be critical for a potential anti-cancer effect remains much discussed, particularly as case studies suggesting a clinical benefit from ascorbate infusions continue to surface (9–17).

Current research is focused on the identification and verification of several proposed mechanisms whereby ascorbate could affect tumor progression. These include: (i) the localized generation of cytotoxic quantities of H_2_O_2_ as a consequence of ascorbate oxidation (15, 18, 19); (ii) ascorbate-dependent activation of the 2-oxoglutarate-dependent dioxygenases that down-regulate the hypoxia-inducible factors (HIFs) (20–22) and that are responsible for the demethylation of DNA and histones (11, 17, 23–25); and (iii) increased oxidative stress induced by dehydroascorbic acid taken up into tumor cells via the glucose transporters (26). These mechanisms all require effective delivery of ascorbate to the tumor.

We have previously measured the diffusion of ascorbate through cell layers in vitro and have modeled its transport through tumor tissue (27). Poor vascularization of tumor tissue not only affects oxygen availability that drives up-regulation of the HIFs, but will also limit the distribution of ascorbate to poorly-perfused regions of the tumor (27). Pharmacokinetic data for plasma ascorbate concentrations from patients treated with high dose infusion are available (4, 6–8) but whether levels of ascorbate in tumor tissue are increased following such treatment in patients has never been reported. We have shown increased tumor ascorbate in a pre-clinical model, with associated decreased expression of HIF-dependent protein markers (28).

We hypothesize that the dysfunctional vasculature of solid tumors results in compromised delivery of ascorbate to poorly vascularized regions of the tumor and that the ascorbate deficit acts as an additional driver of the hypoxic response via HIF upregulation. In this study, we aimed to determine whether supra-physiological plasma concentrations can overcome poor perfusion in solid tumors and whether increasing tumor ascorbate could affect the prevalence of hypoxia markers. Patients with colon cancer were treated with four daily ascorbate infusions immediately prior to surgical resection of their cancer. We measured ascorbate levels in plasma, red blood cells and tumor tissues and the expression of HIF-dependent proteins. As ascorbate can improve surgical wound healing (29) and quality of life for cancer patients (30), we also monitored patient quality of life and recovery following surgery.

## Materials and Methods

### Materials

Unless stated otherwise, all chemicals were from Sigma-Aldrich (St. Louis, MO, USA).

### Ethics and Patient Consent

Ethical approval was obtained from the New Zealand Health and Disability Ethics Committees (15/STH/145). This trial was registered on the Australian New Zealand Clinical Trials Registry (ANZCTR Trial ID ACTRN12615001277538, Universal trial no. U1111-1173-0882). Patients were recruited at Christchurch Hospital, the main tertiary hospital on the South Island of New Zealand. Patients gave informed consent separately to two parts of the study: (i) prior to diagnostic colonoscopy for additional biopsies for tumor and normal tissue; (ii) after cancer diagnosis was confirmed, for randomization to the control arm (no infusions) or intervention arm (4 consecutive days of high dose vitamin C infusions prior to surgery) of the trial. Randomization was carried out by the study coordinator and the recruiting clinician and patient were blinded to this process. Patients declared ethnicity using the New Zealand census question, and an option of sample disposal with karakia (blessing) was offered.

### Selection Criteria

Participant recruitment and selection is shown in **Supplementary Figure 1**. Criteria for inclusion in part 1 (additional biopsies at colonoscopy) were strong clinical suspicion of colorectal cancer and planned confirmatory biopsy. Patients were selected by the attending clinician according to clinical criteria (including anemia, blood in stool or change in bowel habit). If a tumor was found on colonoscopy, the patient was considered for part 2 of the study. Inclusion criteria were (all required): histologically confirmed colon cancer; good functional status (ECOG grade 0 or 1); age ≥ 18 years; adequate bone marrow, hepatic, renal, and cardiac functions. Exclusion criteria were (one required): likely to receive neoadjuvant therapy (e.g., for rectal cancer); serious gastrointestinal disorders including active bleeding; inability to give informed consent; diabetes (high dose ascorbate can have a hypoglycemic effect and interfere with some glucose monitoring tests); regular use of supplements containing ≥ 1 g/day vitamin C; serum creatinine > 175 μmol/L; known erythrocyte glucose-6-phosphate dehydrogenase deficiency (1); serious or uncontrolled infection; cardiac or neurological conditions; pregnant or lactating women; current calcium oxalate nephropathies with the potential to impair urinary flow (31). An adverse response to the test dose of 25 g IV ascorbate given on the first intervention day would also exclude patients from continuing on the study. Adverse events were monitored by the research nurses and clinicians, and recorded using the Common Terminology Criteria for Adverse Events terms and grading (CTCAE, version 4.0).

### Trial Design

The study comprised a therapeutic window of opportunity-style design with an intervention administered between diagnostic colonoscopy and planned surgical resection. Biopsy samples of both normal mucosa and suspected tumor were taken at colonoscopy, and approximate location recorded. A fasting blood sample was also taken. Following colonoscopy, patients who met all inclusion criteria were randomized at a ratio of 3:2 into the infusion or control arm of the study. Patients in the infusion arm received a first dose of 25 g of ascorbate 4 days prior to their planned surgical resection, and then daily ascorbate infusions of up to 1 g/kg body weight (capped at a total daily dose of 75 g) for 3 days (days 2–4). Patients in the control arm did not receive ascorbate infusions, but consented to blood and tissue samples to be taken at resection. Surgery was carried out on day 5. Blood samples were taken daily prior to infusion, and also immediately after infusion in three patients. Patients attended Christchurch Hospital for their planned surgery where the resected specimen was immediately transferred to Anatomical Pathology, Canterbury District Health Board, for pathological review and tissue banking.

### Ascorbate Infusion

Ascor L 500® (McGuff Pharmaceuticals, Inc., Santa Ana, CA), equivalent to 500 mg/ml ascorbic acid, was diluted in 250-ml sterile water for injection (25 g ascorbic acid on day 1) or in 500-ml water (up to 75 g ascorbic acid for days 2–4) immediately prior to use, and infused into the median cubital vein at 0.5–1 g/min. Infusions and monitoring were carried out by experienced research nurses with supervision by an oncologist at Christchurch Clinical Studies Trust. During infusion, the diluted ascorbate was kept at room temperature protected from light. Infusions took between one and two hours on average. Patient vital signs were recorded before, after 10 min and immediately after infusion.

### Quality of Life

Patients completed a validated quality of life and symptom analysis questionnaire (EORTC QLQ-C30) (32) and fatigue questionnaire (MFSI-SF) (33), prior to the first infusion and on the day of the last infusion (the day before surgery).

### Patient Follow-Up

Patients were followed up for 30 days following surgery, with complications recorded using the Clavien-Dindo (CD) score. Length of hospital stay and readmissions were retrieved from medical records. Recurrences, metastases and death were recorded up until 561–745 days post-surgery.

### Blood Samples

Peripheral blood (5 ml) was collected into K_3_-EDTA vacutainer tubes, immediately placed on ice and processed within 2 h. Plasma was removed following centrifugation at 1,000 g for 10 min at 4°C and an aliquot mixed with an equal volume of ice-cold 0.54 M perchloric acid containing 50 µM DTPA. Precipitated protein was removed by centrifugation and supernatants were stored at −80°C. The erythrocytes were prepared for ascorbate analysis, as previously described (34). Briefly, any remaining plasma and the buffy coat layer were removed and the erythrocytes washed with a 10-fold excess of ice-cold PBS containing 500 µM DTPA. A sample of packed red cell suspension (150 µl) was collected following centrifugation at 1,000 g for 10 min at 4°C. Packed cells were stored at −80°C until HPLC analysis. A four times volume of ice-cold milliQ water containing 500 µM DTPA was added to the thawed aliquots and the lysate was spun through a centrifugal filter unit (Amicon Ultra 0.5 ml, 10K Ultracel®, Millipore, Burlington, Massachusetts, USA) to remove hemoglobin. An equal volume of ice-cold 0.54 M perchloric acid containing 50 µM DTPA was immediately added to the ultrafiltrate. Ascorbate content was measured by HPLC as below. The hemoglobin content of the packed cells was determined using Drabkin’s reagent in which hemoglobin is converted to cyanmethemoglobin and the absorbance read at 540 nm after incubation for 20 min at room temperature (35). The intracellular concentration of ascorbate in erythrocytes was calculated by linear regression after taking into account any dilutions and then dividing the measured ascorbate concentration by 0.7 to account for the water content of erythrocytes. To correct for any variation in the packed cell content between samples, the concentrations obtained were adjusted by the (measured vs. expected) hemoglobin content of the packed cell lysate.

### Tissue Samples

During diagnostic colonoscopy, tumor and histologically normal biopsy tissue samples were taken. Normal, uninvolved colon mucosa was sampled at a distance ≥ 10 cm from the tumor. Biopsy material was immediately put on ice and snap frozen in liquid nitrogen within 20 min. At cancer resection, surgical specimens were examined by an expert pathologist, and surplus viable tumor and uninvolved normal tissue at a distance of ≥ 10 cm from the tumor were sampled. Multiple regions of the tumor (edge/periphery, mid and central) were sampled to assess tumor heterogeneity (schematic shown in **Supplementary Figure 2**). Tissue was processed and snap frozen in liquid nitrogen within 45 min of surgical removal and stored at −80°C.

### Tissue Processing for Ascorbate Measurement

Frozen normal bowel and tumor biopsy tissue samples were weighed and homogenized in ice-cold 10 mM potassium phosphate buffer (pH 7.4) in 1.5-ml Eppendorf tubes using a plastic micro tissue homogenizer (Thermo Fisher, Auckland, New Zealand) on ice. Extraction procedures of these samples (10–20 mg) were optimized using mouse breast cancer tissue (University of Otago Animal Ethics Committee approval C01/16). Resection tissue samples (>30 mg) were ground to a fine powder in liquid nitrogen using a pre-cooled mortar and pestle on dry ice as previously described (36). The resulting powder was weighed and homogenized with 10 mM potassium phosphate buffer (pH 7.4) for ascorbate extraction. An equal volume of 0.54 M perchloric acid containing 50 µM DTPA was added to the extracts and samples stored at −80°C until analysis.

### Ascorbate Analysis

Ascorbate content of tissue lysates, plasma and erythrocyte samples was measured by HPLC with electrochemical detection (Thermo Fisher Scientific, Waltham, MA, USA) and quantified against a standard curve (1.25 to 40 μM, prepared daily) (34). Samples were analyzed with and without reduction with 32 mM TCEP for 3 h on ice, which allowed quantification of both ascorbate and dehydroascorbic acid (34). Total ascorbate levels are reported, as dehydroascorbate, determined from the difference between with and without TCEP reduction, was found to be <<10% of the total.

### Tissue Processing for Protein Analyses

Frozen powder from resected tissue was homogenized with 10 mM potassium phosphate buffer for vascular endothelial growth factor (VEGF) ELISA, or with RIPA buffer [50 mM Tris (pH 8), 150 mM NaCl, 1% NP-40, 0.5% sodium deoxycholate, 0.1% SDS, CompleteTM Protease Inhibitor Cocktail (Roche, Basel, Switzerland)] for immunoblot analysis.

### Western Blotting

SDS-PAGE was carried out on tissue homogenates with each sample standardized to 1.5 μg DNA per well. Proteins were separated on 4%–12% Bis-Tris Plus SDS gels and transferred to 0.45 μm polyvinylidene difluoride membranes (Life Technologies, Carlsbad, USA). Membranes were incubated overnight at 4°C with primary antibodies against carbonic anhydrase IX (CA-IX) (1/200, R&D Systems, AF2188), glucose transporter 1 (GLUT1) (1/1,000; Abcam, ab32551), phosphorylated histone protein γH2AX (1/2000; Abcam, ab11174), or ß-actin (1/10,000, Sigma, A5316), and for 1 h at room temperature with secondary horseradish peroxidase-conjugated antibodies (1/5,000, DAKO). β-Actin was used as loading control. For GLUT1, the same positive control (20 µg protein of 1% O_2_ treated T24 cell lysate) was run on each gel to normalize signals between the blots. For CA-IX, a clear cell renal cell carcinoma tissue sample (University of Otago Human Ethics committee H14/020 (37), adjusted to 1.5 μg DNA per well, was similarly run on each gel. For γH2AX, a positive control (WiDr cells from ATCC treated for 4 h with 20 mM ascorbate, 20 µg protein) was run on each gel. Protein bands were visualized using the ECL Prime Western Blotting Detection Reagent (GE Healthcare, Chicago, USA), and quantified with the Alliance 4.7 imaging system and the ImageJ software (36, 37).

### VEGF ELISA

VEGF protein levels were measured in tissue lysates using the human VEGF DuoSET ELISA Development Kit (R&D Systems, DY293B) according to the manufacturer´s instructions.

### Statistics

Data were analyzed with GraphPad Prism version 8.4.2 with significance set at p < 0.05. Ascorbate levels in normal tissue before and after infusion were compared using paired t-tests. Unpaired t-tests and mixed effects models with ANOVA were used to analyze changes in erythrocyte levels and differences in tumor ascorbate and hypoxia marker expression. Pearson’s correlations were used to determine relationships between normal and tumor tissue ascorbate levels and least squares fit was used to determine associations between plasma and tissue ascorbate levels.

## Results

### Patient Accrual and Characteristics

Patients who presented at Christchurch Hospital for a colonoscopy due to suspicion of colon cancer were approached for consent (**Supplementary Figure 1**). Of 53 patients consented for additional biopsies, 15 met all inclusion criteria and consented to the second part of the study. Of the remainder, 30 did not have cancer, four had mid-low rectal cancer and likely to receive neoadjuvant therapy, three consented to biopsy only and one withdrew prior to biopsy. Of the 15 enrolled study participants, nine patients were randomized to the vitamin C infusion arm and six to the control arm. The control cohort was all men, whereas the infusion cohort contained three women and six men. The average age was similar in both cohorts and ethnicity was predominantly European.

### Tumor Characteristics

Clinicopathological data for the study participants is shown in **Table 1**. Tumor stages I–III/IV were represented in both cohorts, with variable presentation of positive nodes, vascular/perineural invasion, and the presence of infiltrating lymphocytes (**Table 1**).

**Table 1:** Patient and clinicopathological data.

Parameter		Control	Infusion
		*n* = 6	*n* = 9
**Gender**	Female, male	0, 6	3, 6
**Age**	Mean ± SD (years)	74 ± 6	72 ± 8
***Pathology data:***			
**AJCC stage**	I and II	4	4
	III and IV	2	5
**Grade**	Well differentiated	3	0
	Moderate diff/low grade	3	9
	Poor diff/high grade	0	0
**Tumor size**	Mean ± SD (mm)	46 ± 28	37 ± 11
**Lymph node**	Positive	0	4
**Vascular/perineural invasion**	Positive	1	6
**Infiltrating lymphocytes**	Positive	1	5
***Follow-up observations:***			
**Average length of hospital-stay**	Mean ± SD (days)	9.3 ± 5.4	5.8 ± 2.4
**Readmission within 30 days**		2	1
**Surgical complications**	No. of patients	3	3
**Clavien-Dindo**	Grade I (episodes, patients) Grade II (episodes, patients) Grade III (episodes, patients)	2, 2 7, 3 0, 0	1, 1 2, 2 2, 1
	Grade IV/V (episodes, patients)	0, 0	0, 0
**Metastasis**		1	1
**Death within 2 years**		2	1

### Plasma Ascorbate Levels and Effects of Infusion

**Figure 1:**
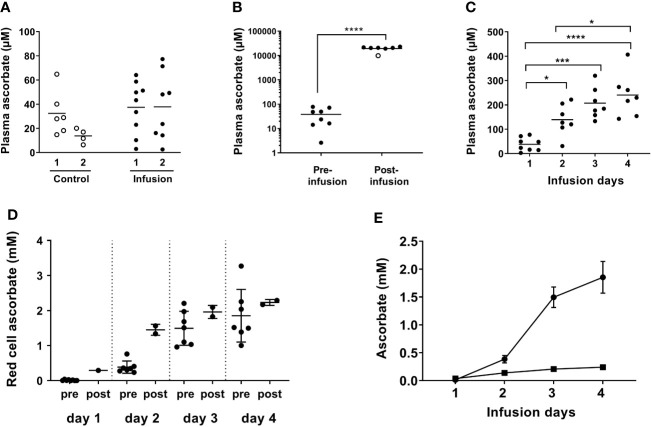
Plasma and red blood cell ascorbate levels in patients at baseline, immediately post-infusion and over the course of high dose ascorbate infusion. IVC was administered daily for 4 days and blood taken pre- and post-IVC infusion as indicated. The first infusion comprised 25 g ascorbate and subsequent infusions were at 1 g/kg body weight, with a maximum of 75 g. **(A)** Baseline plasma ascorbate from patients in the control (○) and infusion (⬤) cohorts at colonoscopy (1) and at surgery (2) (control patients) or immediately prior to first infusion (Infusion patients). Horizontal bars show the mean value. **(B)** Plasma ascorbate in samples taken prior to first infusion and immediately after infusion with high dose ascorbate. Horizontal bars show the mean value. (○) sample following 25 g infusion. ****p < 0.0001 (unpaired t-test). **(C)** Plasma ascorbate immediately prior to consecutive high dose ascorbate infusions on days 1–4. Horizontal bars show the mean value. Significant differences were observed between day 1 plasma ascorbate and the subsequent days ***p < 0.005, ****p < 0.0001 (unpaired t-tests). There was a significant difference between concentrations at days 1 and 4, *p < 0.05 and a one-way ANOVA indicated a significant difference between days 1 and 4, p < 0.0001. **(D)** Red cell ascorbate concentrations pre- and post-infusion; means ± SD. Unpaired Student’s t test showed no significant differences between red cell ascorbate concentrations on day 2 post-infusion and pre-infusion day 3, and post-infusion day 3 and pre-infusion day 4. **(E)** Plasma (▪) and red cell (⬤) ascorbate concentrations measured prior to infusion each day; means ± SD. A mixed-effects model indicated a significant difference between days, with day 1 different to day 2, 3, and 4; p < 0.005 for red cells and p < 0.05 for plasma.

Prior to ascorbate infusion, fasting plasma ascorbate concentrations were similar for both control and infusion groups. Levels ranged from 3.0 to 64.8 µM ascorbate, with means ± SD of 32.5 ± 18.2 µM for control and 37.4 ± 21.5 µM for infusion patients (**Figure 1A**). At diagnostic biopsy baseline, two patients were defined as ascorbate deficient and at risk of scurvy (<11 µM), two were marginally deficient (11–23 µM), seven inadequate (23–50 µM) and four adequate (>50 µM), with cut-offs as previously defined (38). Upon repeated measurement prior to surgery, or before first infusion, an intervening period of 31 days (median, range 5–90 days), average plasma levels remained low, with 7/12 patients now classified as ascorbate deficient or marginally deficient, compared with 4/12 at diagnostic presentation (**Figure 1A**).

Plasma ascorbate monitoring in three patients immediately after infusion indicated that levels reached ~10 mM following the first infusion of 25 g ascorbate and ≥ 20 mM following subsequent infusions at 1 g/kg or 75 g maximum (**Figure 1B**). At 24 h post-infusion, most of the ascorbate had been cleared and plasma levels had returned to the micromolar range as expected. However, baseline plasma levels increased steadily over the four infusion days and were above the 100 µM normal saturating level in the days following infusions (**Figure 1C**). These daily levels increased significantly from 38 ± 28 µM on day one, to 139 ± 65 µM on day two, to 207 ± 65 µM on day three, to 241 ± 88 µM on day four prior to the final infusion (p < 0.001) (**Figure 1C**).

### Erythrocyte Ascorbate Levels and Effects of Infusion

Mature red blood cells do not express the specific vitamin C transporters that allow active ascorbate accumulation against a concentration gradient (39). Consequently, ascorbate levels in red blood cells reflect passive accumulation or uptake of dehydroascorbate via the glucose transporters (40), and are generally similar to plasma concentrations (34). Ascorbate concentrations in erythrocytes at biopsy were 18 ± 20 µM, compared to 38 ± 28 µM in matching plasma. Erythrocyte ascorbate increased significantly immediately post-infusion (**Figure 1D**) and levels continued to increase over the four-day infusion period (p < 0.005). In contrast to results with plasma, erythrocyte levels were sustained at post-infusion peaks for 24 h, with the ascorbate concentration measured pre-infusion equivalent to post-infusion on the previous day (**Figure 1D**). Therefore, the erythrocyte ascorbate concentration accumulated to levels significantly higher than in plasma, with average concentrations of ~2 mM on days 3 and 4 compared to ~0.2 mM in plasma (**Figure 1E**).

### Tissue Ascorbate Levels

**Figure 2:**
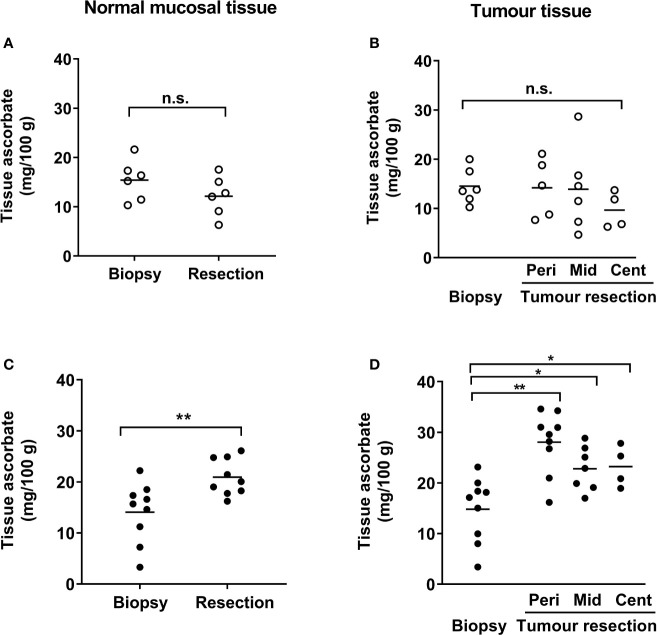
Ascorbate levels in patients with colorectal cancer in normal mucosa and in tumor tissue. Tissue levels in control **(A, B)** and infusion patients **(C, D)**, with samples taken at diagnostic colonoscopy and after resection. Ascorbate levels were unchanged in normal mucosa **(A)** and in tumor tissue **(B)** from control patients, but were significantly increased in both normal mucosa **(C)** and tumor tissue **(D)** following infusions. Tumor lesions were sampled at multiple locations as follows: edge/periphery (Peri), mid-tumor (Mid) and central core (Cent). Not every part of the tumor was sampled in every patient and hence sample numbers may vary for the tumor regions **(B, D)**. Individual data for control (*n* ≤ 6) and infusion cohorts (*n* ≤ 9) with means are shown. Results were compared using paired t-tests for changes following infusion, as shown. *p < 0.05, **p < 0.01, n.s., not significant.

Tumor biopsy samples taken during colonoscopy showed similar levels of ascorbate for both normal mucosa and tumor tissues in the control and infusion cohorts (**Figures 2A, C** and **Table 2**). At resection, ascorbate levels in normal mucosa or in tumor tissue were unchanged in the control cohort (**Figures 2A, B** and **Table 2**). In contrast, with ascorbate infusion over 4 days, both tumor and normal tissue ascorbate increased significantly (**Figures 2C, D**). Ascorbate in tissue from the tumor periphery appeared higher than tissue from mid or central regions in both control and infusion samples (**Figures 2B, D** and **Supplementary Figures 2A, B**). Tumor ascorbate increased significantly post-infusion and was higher than in tumors from the control cohort, regardless of whether the samples were derived from peripheral, middle or central locations within the tumor (**Figures 2B, D** and **Table 2**).

**Table 2:** Ascorbate levels in normal mucosal bowel tissue and tumors pre- and post-ascorbate infusions.

Sample	Ascorbate content (mg/100 g tissue)		
	Control Cohort (*n*)	Infusion cohort (*n*)	Significance
**Normal mucosa:**			
Biopsy Resection	15.4 ± 4.1 (6) 12.1 ± 4.0 (6)	14.1 ± 5.9 (9) 20.9 ± 3.6 (9)	0.662 0.0006***
**Tumor:**			
Biopsy	14.5 ± 3.6 (6)	14.8 ± 6.4 (9)	0.925
Resection			
Periphery Mid-tumor Central	14.9 ± 5.9 (5) 13.9 ± 8.5 (6) 9.7 ± 3.7 (4)	28.1 ± 6.1 (9) 22.8 ± 4.4 (7) 23.2 ± 4.1 (4)	0.0014** 0.033* 0.0026**

Results show means ± SD for samples from (n) individual patients. Significant differences are recorded for unpaired t-tests between data from the control and infusion cohorts, with higher levels being recorded in normal mucosa and tumor tissue (periphery, mid and central regions) in the infusion cohort. *p < 0.05; **p < 0.01; ***p < 0.001. Bold values are statistically significant.

**Figure 3:**
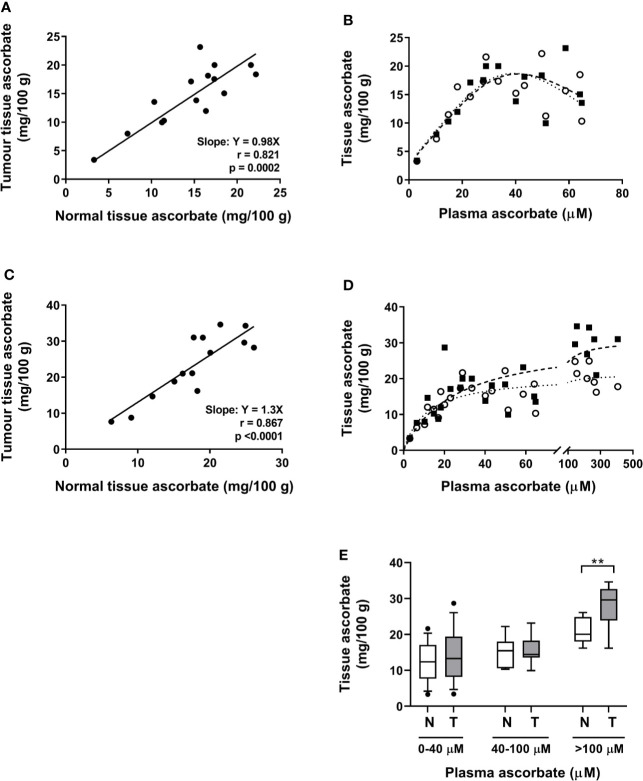
Associations between tumor and normal tissue ascorbate, and between tissue and plasma ascorbate levels in patients with colon cancer. **(A)** At biopsy, tumor and normal mucosa tissue ascorbate is closely correlated (Pearson r = 0.82, p = 0.0002). **(B)** Association between plasma ascorbate levels and normal mucosa (○) and tumor (▪) tissue in patients at biopsy (least squares fit, R^2^ for best fit 0.64 and 0.62 for normal (^…….^) and tumor (—), respectively). **(C)** At resection, a correlation between tumor periphery and normal tissue is seen (Pearson r = 0.87, p < 0.0001). **(D)** Association between plasma levels (pre-infusion on day prior to resection) and normal mucosa (○) and tumor periphery (▪), with non-linear saturation curve fit indicated (R^2^ for best fit 0.85 and 0.69 for normal and tumor, respectively). Individual data for control (*n* = 6) and infusion cohorts (*n* = 9). **(E)** Comparison of normal mucosa (N) and tumor (T) tissue ascorbate levels with increasing plasma levels. Box plots show medians and the interquartile range with whiskers representing the 10–90 percentiles. There is a significant difference between mucosal and tumor tissue when plasma levels exceed the normal physiological maximum of 100 µM (unpaired t-test, **p = 0.008).

There was a close correlation between ascorbate levels in tumor tissue and adjacent normal tissue in all cases, at baseline and following resection (Pearson r = 0.821, p = 0.0002 and r = 0.867, p < 0.0001 for baseline and resection tissue, respectively) (**Figures 3A, C**). These data are for measurements from the tumor periphery. Similar correlations were also seen between normal tissue and other parts of the tumor (mid-tumor R^2^ 0.56, p = 0.003, tumor centre R^2^ 0.70, p = 0.01).

When data from both patient cohorts are combined, a non-linear saturation curve is evident which is similar for normal and tumor tissue, by plotting plasma levels on the day of biopsy vs. biopsy tissue levels in normal mucosa and tumor periphery (**Figure 3B**). There was a strong linear relationship between plasma and tissue levels (tumor and normal) at plasma ascorbate concentrations below 40 µM (**Figure 3B**). Above these levels, the association between plasma and tissue ascorbate followed a non-linear saturation curve (least squares fit, R^2^ = 0.64 and 0.62 for normal and tumor, respectively (**Figure 3B**). At resection, a similar linear relationship between plasma levels, taken on the day prior to surgery, and tissue levels was seen at lower plasma levels (least squares fit, R^2^ = 0.85 and 0.69 for normal and tumor periphery, respectively, **Figure 3D**). However, ascorbate infusions affected tumor and normal tissue differently at higher ascorbate concentrations. Increasing plasma ascorbate in the physiological range (up to 100 µM), affected both normal mucosa and tumor tissue equally, whereas tumor levels increased more readily than normal mucosal tissue levels when concentrations exceeded 100 µM (**Figures 3B, D, E**).

### Expression of HIF-Dependent Proteins and Markers of Oxidative Stress Following Infusion

**Figure 4:**
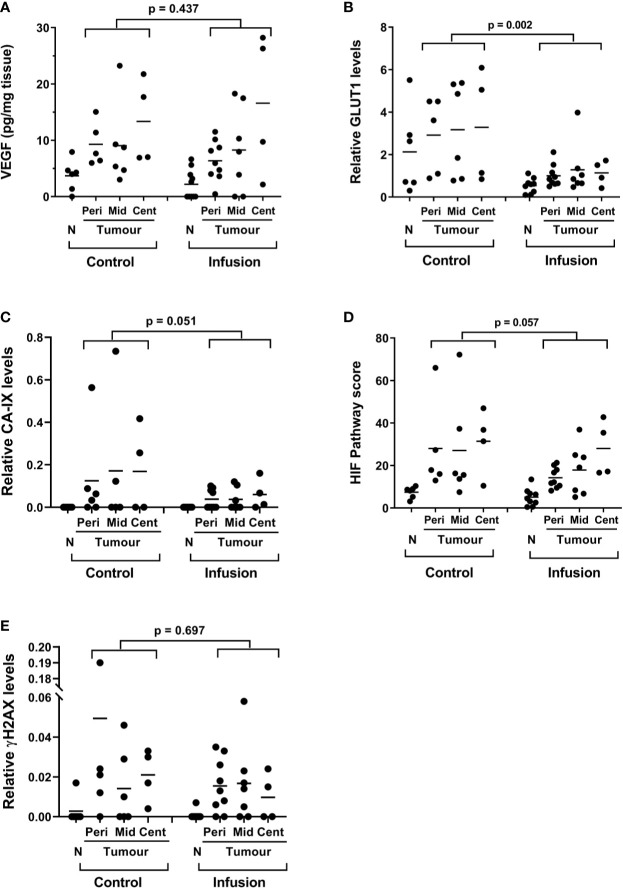
Protein expression associated with the hypoxic response and oxidative stress response in bowel mucosal tissue and in tumor tissue in control and infusion cohorts. Relative levels of **(A)** VEGF, **(B)** GLUT1, **(C)** CA-IX, were monitored in normal mucosal and tumor tissue from each individual. Expression of each protein was elevated in tumor tissue relative to control. Levels were lower in tumor tissue (all tumor regions) from the infusion cohort when compared with the levels in control tumor tissue (unpaired t-test results shown). **(D)** HIF Pathway score was derived from the combined measurement of the three proteins for each tumor sample. **(E)** Relative levels of γH2AX in tumor and normal mucosal tissues. Comparative statistics for combined tumor samples from peripheral, mid and central regions of the tumors are shown (unpaired t-tests). In addition, one way ANOVA with fitting for a mixed effects model (Brown-Forsythe and Welch test) indicated a significant increase in the tumor regions from periphery to central for the combined HIF Pathway score (p = 0.02).

We monitored the levels of VEGF, GLUT1 and CA-IX as markers of HIF activation in the surgical resection samples, as previously (22, 37) and have compared tumor and adjacent normal mucosal tissue in control and infusion patient samples. Hypoxic marker protein levels were measured by western blotting (GLUT1, CA-IX) (**Supplementary Figure 3A**) and by ELISA (VEGF). Expression of GLUT1, CA-IX and VEGF was significantly higher in tumor tissue compared with normal mucosal tissue (**Supplementary Table 1** and **Figures 4A–C**). When comparing post-infusion tumor tissues with control tumors, HIF-associated proteins expression appeared to be lower in tumors following infusion, with a significant difference in GLUT1 levels (p = 0.002) and a strong trend in CA-IX (p = 0.051) (**Figures 4A–C**). To determine whether ascorbate impacted HIF transcriptional activity in the tumor tissue, we derived a HIF pathway score for each tumor sample by normalizing the relative expression values for each protein as percent expression of the highest sample and combining the GLUT1, CA-IX, and VEGF scores for each sample. There was a trend for a lower HIF pathway score in the post-infusion tumor samples than in the control tumor tissue (**Figure 4D**; p = 0.057). There also was a significant negative correlation between tumor ascorbate levels and VEGF expression (r = −0.383, p = 0.023) and the HIF Pathway score (r = −0.430, p = 0.01) (**Supplementary Figure 4**).

Levels of the phosphorylated histone DNA repair protein γH2AX were measured as a marker of oxidative DNA damage (41). Colorectal cancer cells (WiDr) treated *in vitro* with 20 mM ascorbate for 2 h or 4 h showed a robust γH2AX immunoreactive band (**Supplementary Figure 3B**), and were used as positive control for the tissue samples. Measured levels of γH2AX were very low in tumor tissue and generally undetectable in normal mucosal tissue (**Supplementary Table 1** and **Supplementary Figure 3C**). There was no difference in levels detected between the post-infusion and control cohorts (p = 0.697) (**Figure 4E**).

### Adverse Events and Quality of Life

Ascorbate infusion was carried out at 0.5 g/min and increased to 1 g/min after 10 min if tolerated, but decreased if any discomfort was noted or any adverse symptoms occurred. Adverse events were Grade 1 according to CTCAE, version 4.0; asymptomatic or mild symptoms, and intervention was not indicated. Three of nine patients reported no discomfort or adverse events. For 5/36 infusions, infusion flow rate was reduced from 1 g/min to 0.75 g/min or 0.5 g/min due to increased blood pressure or tingling in fingers. Elevated blood pressure was noted on 9/36 infusions, which all resolved post-void (**Supplementary Figure 5**), which is consistent with published observations (6, 13). Three events of tingling fingers (transient) and three of light-headedness (transient) were reported. Otherwise, data on patient vital signs during infusions were unremarkable (**Supplementary Figure 5**). Quality of life was recorded at colonoscopy and prior to resection, showing universally high functional scores, low symptom scores, and low fatigue with high vigour scores in this cohort. These levels did not change following the course of four high dose ascorbate infusions (**Supplementary Figure 6**).

### Follow-up

While the trial was not powered to determine clinical efficacy, patients were followed up for the first 30 days and then up to two years post-surgery (**Table 1**). Patients in the control cohort tended to have a longer hospital stay post-surgery (9.3 days vs. 5.8 days, p = 0.105). Surgical complications were all minor (CD 1-2 complications) except for one patient in the infusion arm who suffered an anastomotic leak and subsequent enterocutaneous fistula (CD Grade III). One patient in each cohort developed metastases to the liver. Two patients in the control group died at nine months and twelve months and one in the infusion cohort at nine months (**Table 1**).

## Discussion 

Our window-style clinical intervention study, has provided detailed information on the uptake of ascorbate into tumor tissue following high dose ascorbate infusions. Whether this treatment affects tumor ascorbate content has not previously been reported and this is important when determining the potential for clinical efficacy. While often discussed (9, 42) and raised in clinical studies (8, 13, 16, 43) there has been no information on whether using high dose infusions with cancer patients affects tumor ascorbate levels or biology. Our novel data show that ascorbate infusion over 4 days significantly increased both normal mucosa and tumor ascorbate levels in patients with colon cancer. This is consistent with predictions from modeling ascorbate diffusion uptake through multicellular tissue layers that suggested that variable plasma ascorbate concentrations would be reflected in variable tissue levels (27). Indeed we noted a linear relationship between both normal mucosa and tumor tissue ascorbate and plasma levels when concentrations were below saturation. It appears that tumor tissue accumulated higher ascorbate concentrations than normal mucosa when plasma levels were elevated beyond saturation, as was apparent in the post-infusion samples. This suggests that there may be an advantage in supplying ascorbate at higher concentrations than can be achieved by dietary intervention alone. However, our study does not determine whether the same effect on tumor content could have been achieved with lower infusion doses of ascorbate as predicted by our modeling study (27). This would require further testing.

Plasma ascorbate concentrations were below average for most of our cohort at recruitment, and generally decreased further in the period between diagnosis and surgery. This is not surprising as increased prevalence of ascorbate insufficiency in cancer patients has also been noted by others (44, 45). Peak plasma levels achieved following infusion were similar to those reported by others (6, 7) and clearance of these high millimolar levels occurred over the following 24 h, as expected. It was of interest that the baseline plasma levels increased daily over the 4-day infusion period and were maintained above 100 µM. This may reflect the accumulation of ascorbate in erythrocytes, where levels were seen to increase to millimolar levels following infusion and to be maintained at these concentrations for the following 24 h. Given that red blood cells are a major compartment of whole blood, they may represent a significant ascorbate pool of ascorbate that could be available via leakage from the cells to buffer plasma levels over the infusion period, resulting in continually elevated baseline plasma status. Red blood cell ascorbate levels are known to closely match plasma levels (34, 46). Here, we report that erythrocyte ascorbate concentrations remain elevated following infusion, with potential effects on plasma status. This finding deserves consideration when determining an effective dosing regimen for ascorbate by infusion.

Our data suggest that achieving supraphysiological plasma ascorbate concentrations could be advantageous for the delivery of ascorbate to tumor tissue. We have proposed that ascorbate delivery will be compromised in poorly perfused areas (27) and the data from our study showing notably lower ascorbate levels in central tumor than in peripheral regions are consistent with this prediction. Elevation of plasma ascorbate by infusion could overcome the challenge of delivery to poorly vascularized core regions of solid tumors by increasing the efficacy of delivery (47). Our previous modeling data indicated that delivery to all areas of the tumor would occur when plasma ascorbate levels approached 1 mM. These levels are readily achievable via infusion (8, 13, 16, 43). Our data also indicate that the generation of an ascorbate reservoir in erythrocytes could contribute to sustained elevated plasma levels.

The tissue protein expression levels showed a similar inverse relationship between ascorbate and markers of hypoxia as we have previously observed in retrospective analyses of human endometrial, breast, renal and colorectal tumor samples (22, 36, 37, 48). A similar finding was described in thyroid cancer (49). Biopsy material was insufficient for the protein expression analysis, and only comparisons between the control and infusion cohorts were possible, but these suggest that HIF activation may be moderated by elevating tumor ascorbate. The relationship between ascorbate and HIF-1 has been investigated in pancreatic tumor cells *in vitro*, with millimolar pharmacological concentrations of ascorbate initiating cytotoxicity while decreasing HIF-1 activation (50). Cytotoxicity induced by millimolar ascorbate concentrations in cell culture conditions was associated with elevation of the phosphorylated histone protein γH2AX, signalling a response to DNA damage initiated by H_2_O_2_ generated in the medium (51–53) and is also observed following radiation treatment (41, 51). Consistent with these studies, we readily detected γH2AX in cultured cells incubated with pharmacological ascorbate, but this was not replicated in the patient-derived tumor tissue following HDVC infusions. That there was no difference between the γH2AX levels in tumors from the control or infusion cohorts suggests that ascorbate levels following infusion did not reach concentrations sufficient to induce an oxidative stress in these tissues. It has also previously been noted that oxidative damage by ascorbate is limited in a hypoxic environment (54) and this may impact on the likelihood of intra-tumor oxidative stress initiated by ascorbate. These findings require validation in a much larger cohort that was beyond the scope of the current study.

None of the patients in the infusion cohort reported significant adverse events (> Grade 1) associated with the infusion and there was little change in quality of life, supporting prior safety data for this vitamin. Our prior observational studies showed that higher tumor levels of ascorbate were associated with improved disease-free and disease-specific survival in colorectal and breast cancer (22, 36), but this intervention trial was not powered to address treatment efficacy.

In summary, our study has provided a rich dataset and robust measurements acquired for each patient, with matched plasma, normal and tissue data both before and after an intervention with high dose ascorbate by infusion. This is the first demonstration that tumor ascorbate levels are significantly increased following high dose ascorbate infusion, which fills a fundamental gap in researching the claimed biological effects of ascorbate infusions in patients with (colon) cancer.

### Conclusions

Many patients with cancer access high dose vitamin C infusions via complementary medicine providers, but evidence of efficacy is lacking. Our study of patients with colon cancer shows that ascorbate concentrations are lower in the tumor core than in the periphery. Administration of ascorbate by intravenous infusions over 4 days prior to surgical resection resulted in supra-physiological plasma concentrations and compartmentalization of high ascorbate concentrations into erythrocytes. Ascorbate levels increased in all tumor regions, suggesting that increased plasma availability ensured effective accumulation throughout the tumor. Markers of tumor hypoxia were lower in the post-infusion tumors compared with the control cohort, which suggests that increasing ascorbate levels could moderate the activation of the hypoxia-inducible factors. Our data provide a rationale for further investigation into the potential advantages for the targeted use of vitamin C infusions, rather than oral supplementation, in patients with solid tumors.

## Data Availability Statement

The original contributions presented in the study are included in the article/**Supplementary Material**. Further inquiries can be directed to the corresponding author.

## Ethics Statement

The studies involving human participants were reviewed and approved by New Zealand Health and Disability Ethics Committees (15/STH/145). The patients/participants provided their written informed consent to participate in this study.

## Author Contributions

JG consented patients and performed many of the colonoscopies and operations. HM coordinated sample collection. AC obtained ethical approval, coordinated the study, and carried out quality of life assessments. CW and SB processed and analyzed samples. JP coordinated and provided oversight of all ascorbate analyses and analyzed erythrocyte levels. TE and BR were responsible for patient safety and clinical oversight. GD and MV designed, co-ordinated, and managed the study analyzed the data, and wrote the manuscript. All authors contributed to the article and approved the submitted version.

## Funding

Financial support for this study was obtained from the following: Health Research Council of NZ (15/541), Vitamin C for Cancer Trust (University of Otago), Mackenzie Charitable Foundation (GUD), University of Otago Doctoral Scholarship (CW), Health Research Council of NZ Hercus Fellowship (ACC), and Cancer Society Canterbury West Coast Division for support of the Tissue Bank Christchurch. None of the funders played any role in the design of the study and in the collection, analysis, and interpretation of data and in writing the manuscript.

## Conflict of Interest

The authors declare that the research was conducted in the absence of any commercial or financial relationships that could be construed as a potential conflict of interest.

## Abbreviations

CA-IX, Carbonic anhydrase IX; CD, Clavien-Dindo; CTCAE, Common Terminology Criteria for Adverse Events terms and grading; EORTC-QLQ, European Organization for Research and Treatment of Cancer Quality of Life Questionnaire; GLUT1, Glucose transporter 1; γH2AX, Phosphorylated histone protein. HIF, Hypoxia-inducible factor; HPLC, High-performance liquid chromatography; MSFI-SF, Multidimensional Fatigue Symptom Inventory-Short Form; RCT, Randomized controlled trial; TCEP, Tris(2-carboxyethyl)phosphine hydrochloride; VEGF, Vascular endothelial growth factor.
