# Supplementary material for: Vitamin C Administration by Intravenous Infusion Increases Tumor Ascorbate Content in Patients With Colon Cancer: A Clinical Intervention Study
Source: Front Oncol. 2021 Jan 11;10:600715. doi: 10.3389/fonc.2020.600715 (PMC7830882; doi:10.3389/fonc.2020.600715)
Supplement: Supplementary file 1 [file DataSheet_1.docx]

Supplementary Material

Vitamin C administration by intravenous infusion increases tumor ascorbate content in patients with colon cancer: a clinical intervention study

Gabi U Dachs^1^*, Jamish Gandhi^2^*, Christina Wohlrab^1^, Anitra C Carr^3^, Helen R Morrin^1,4^, Juliet M Pullar^5^, Simone B Bayer^5^, Tim W Eglinton^2^, Bridget A Robinson^1,6^, Margreet CM Vissers^5^

*These authors contributed equally

^1^Mackenzie Cancer Research Group, Department of Pathology and Biomedical Science, University of Otago Christchurch, NZ.

^2^Department of Surgery, Christchurch Hospital, University of Otago Christchurch, NZ.

^3^Nutrition in Medicine Research Group, Department of Pathology and Biomedical Science, University of Otago Christchurch, NZ.

^4^Cancer Society Tissue Bank, University of Otago Christchurch, NZ.

^5^Centre for Free Radical Research, Department of Pathology and Biomedical Science, University of Otago Christchurch, NZ.

^6^ Canterbury Regional Cancer and Haematology Service, Canterbury District Health Board, and Department of Medicine, University of Otago Christchurch, NZ.

# Supplementary Figures and Tables

**
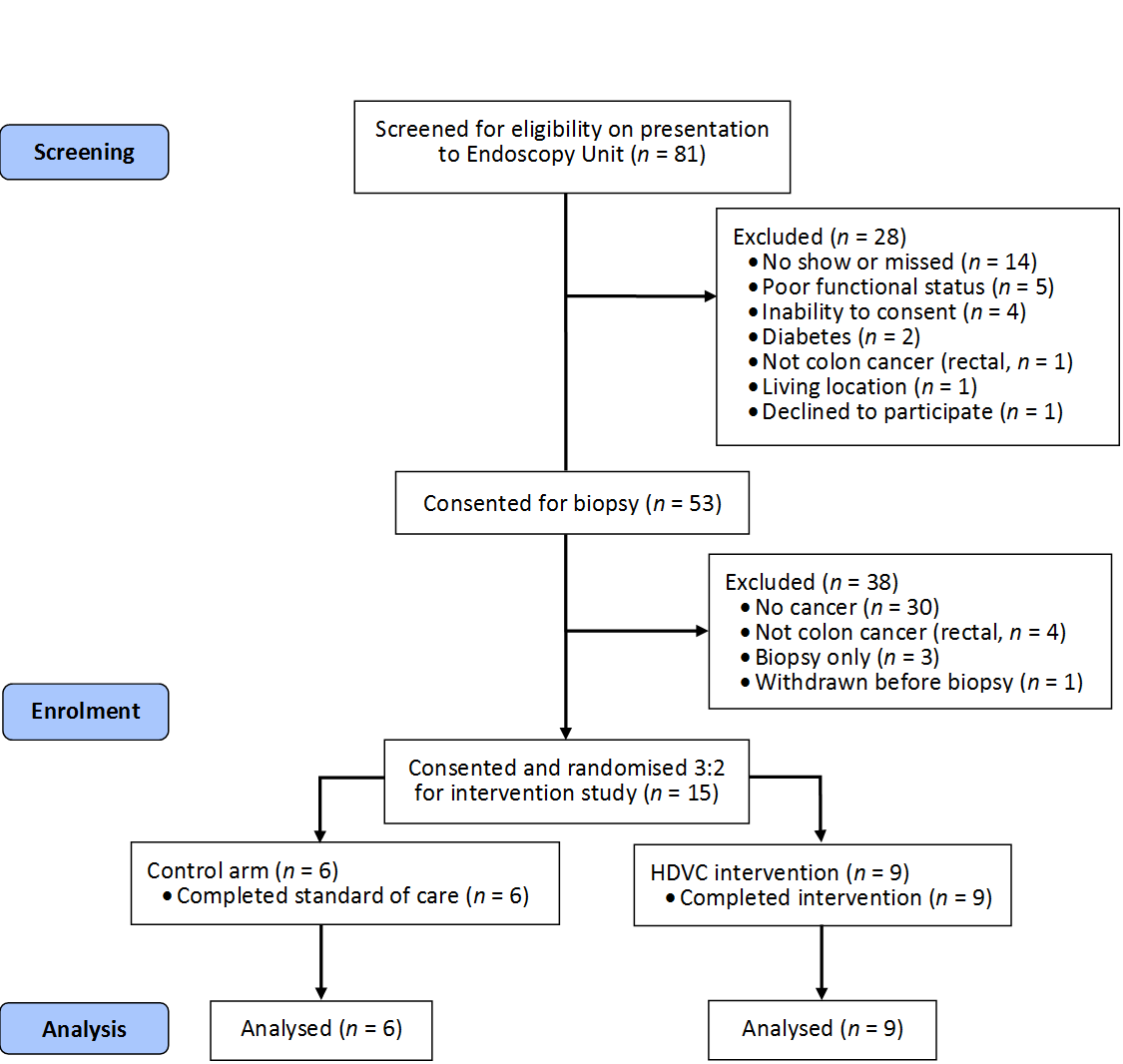
**

**Supplementary Figure 1.** Participant and recruitment flow chart.


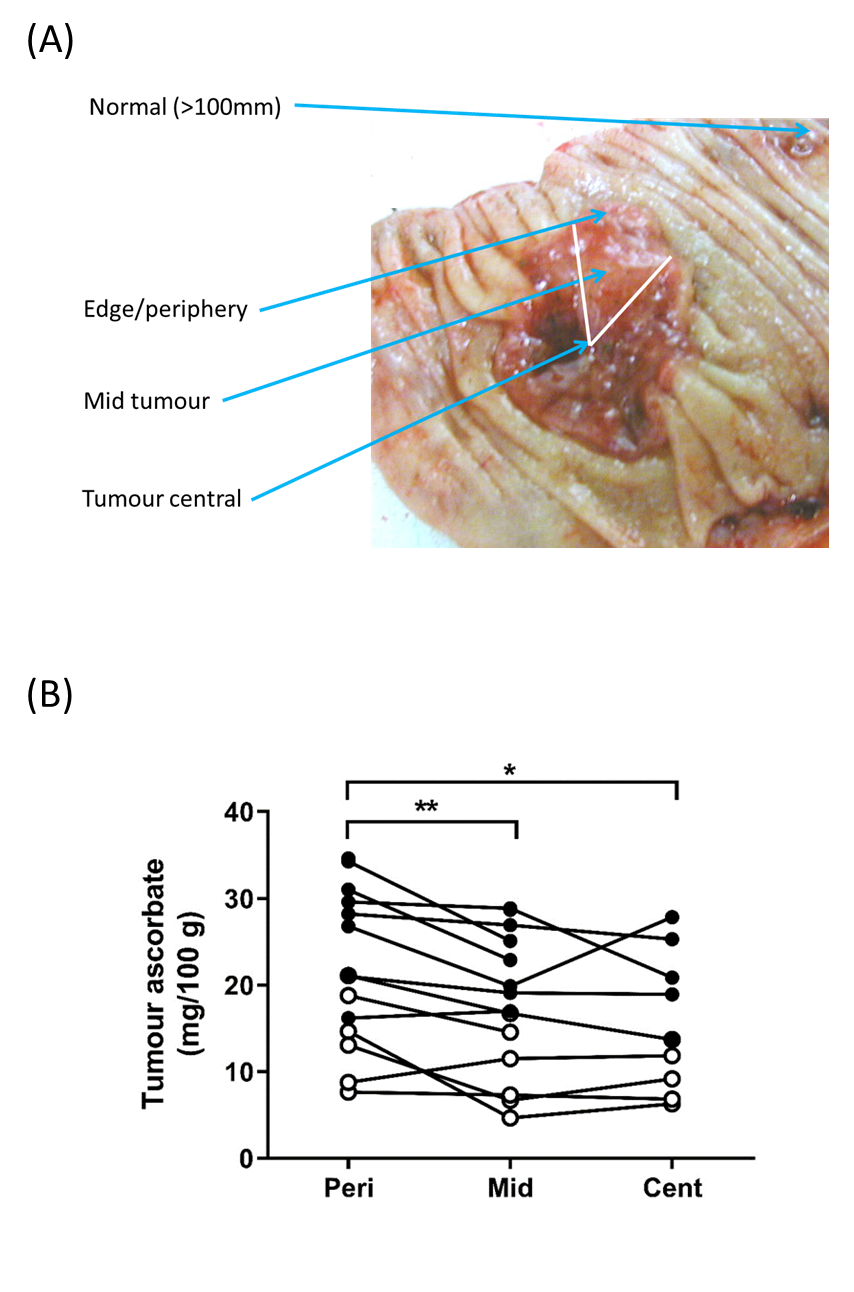


**Supplementary Figure 2.** (A) Schematic showing the regions sampled across a colon cancer lesion as well as sampling from normal mucosa. (B) Ascorbate levels in periphery, mid and central tumor regions. Levels were significantly lower in the mid and central regions than in the periphery for both control (⭘) and post-infusion (⚫) tumors. Results from individual patient tissues are shown. **p < 0.01, *p < 0.05


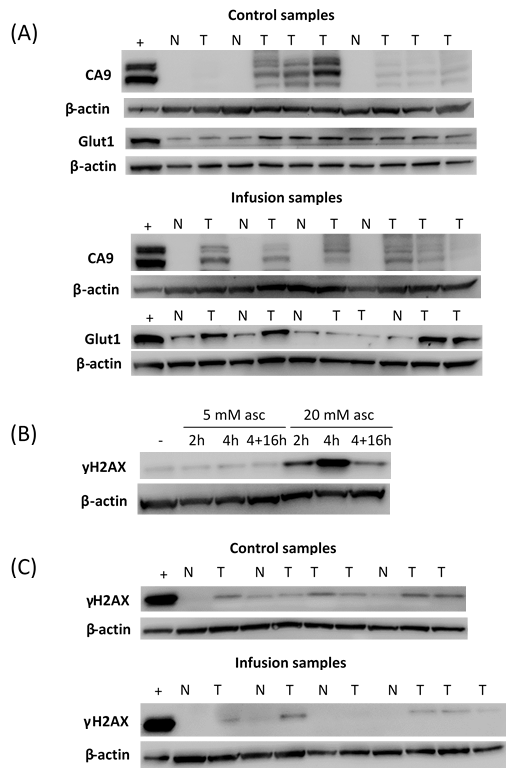


**Supplementary Figure 3.** Western blotting exemplars. (A) HIF-dependent proteins in tissue samples from control and ascorbate infusion patients with colon cancer. (B) DNA damage marker gH2AX in WiDr colon cell line following exposure to 5 mM or 20 mM ascorbate for 2 h or 4 h, or 4h followed by 16 h recovery. (C) γH2AX in tissue samples from control or infusion patients. +, H_2_O_2_ treated WiDr cells positive control; N, normal mucosa; T, tumor; β-actin was used as loading control.

**
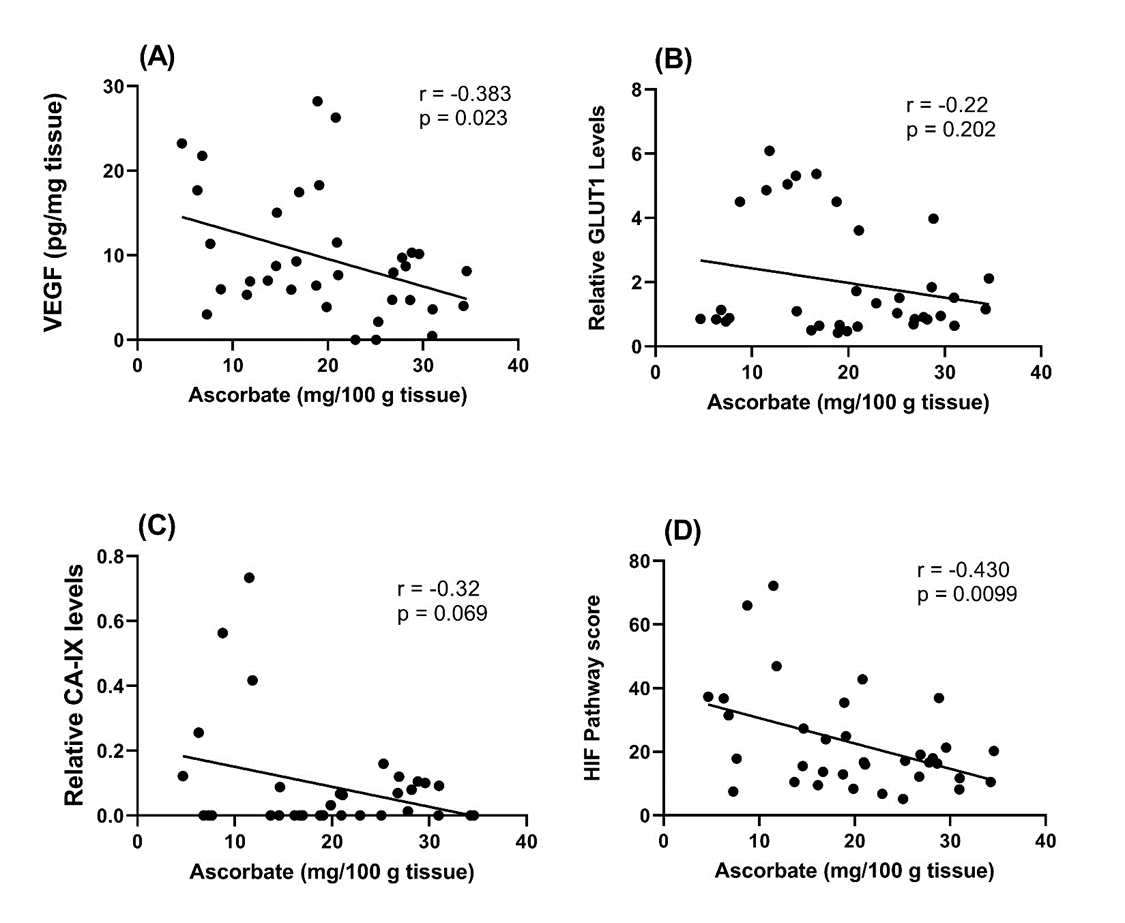
**

**Supplementary Figure 4.** Pearson’s correlations between expression of HIF-dependent proteins (A) VEGF, (B) GLUT1, (C) CA-IX) and (D) the derived HIF Pathway score with tumor ascorbate levels. All tumor data from control and infusion cohorts were included in this analysis.


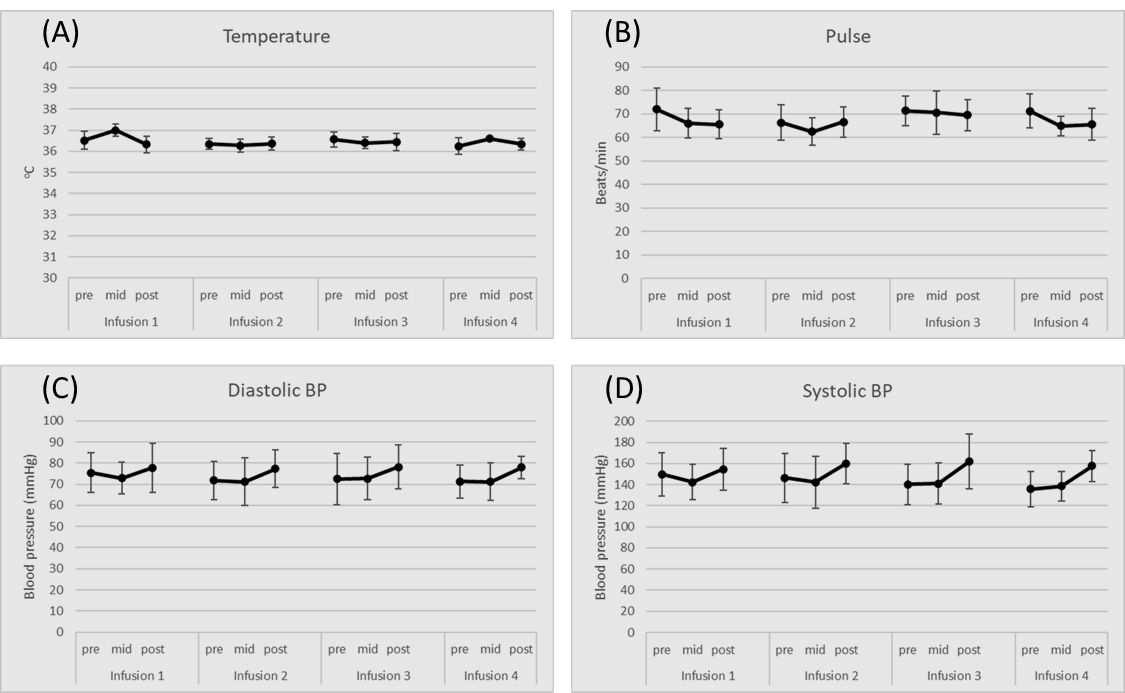


**Supplementary Figure 5.** Vital signs of patients with colon cancer during high dose ascorbate infusions, showing changes in average body temperature (A), pulse rate (B) and blood pressure (diastolic (C) and systolic (D)) at measurements at pre-infusion, at 10 min into infusion and after infusion with up to 1 g/kg ascorbate on days 1-4. Mean ± SEM, *n* = 9.

**
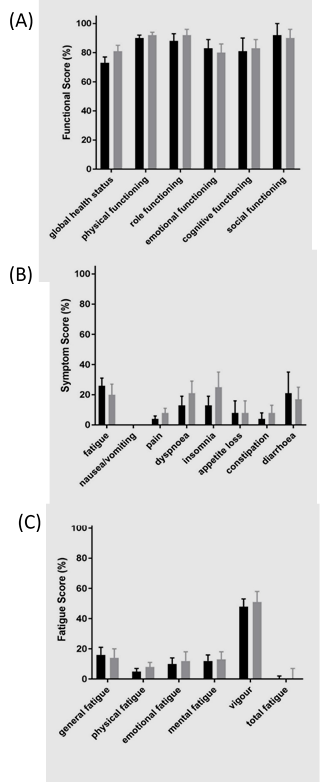
**

**Supplementary Figure 6.** Quality of life scores according to the EORTC QLQ-C30 and MFSI-SF questionnaires of patients with colon cancer in the infusion cohort. (A) Functional score, (B) symptom score and (C) fatigue score. Black bars are at baseline, grey bars before surgery, after all high dose ascorbate infusions; mean ± SD, *n* = 9.

**Supplementary Table 1.** Protein expression levels in matched normal and tumor tissues from all study participants.

| Sample | Normal mucosa | Tumor tissue | Significance |
| --- | --- | --- | --- |
| VEGF  (pg/mg tissue) | 0.823 ± 0.209 (14) | 1.534 ± 0.211 (14) | **0.0031**** |
| GLUT1  (Relative protein levels | 1.176 ± 0.376 (15) | 1.687 ± 0.385 (15) | **0.023*** |
| CA-IX  (Relative protein levels) | 0 ± 0 (14) | 0.073 ± .037 (14) | **0.0078**** |
| γH2AX  (Relative protein levels) | 0.002 ± 0.001 (14) | 0.028 ± 0.013 (14) | 0.068 |

Results show means ± SE for samples from (*n*) individual patients. Significance levels are recorded for paired t-tests between data from the normal mucosa and tumor tissue from each individual patient.

**
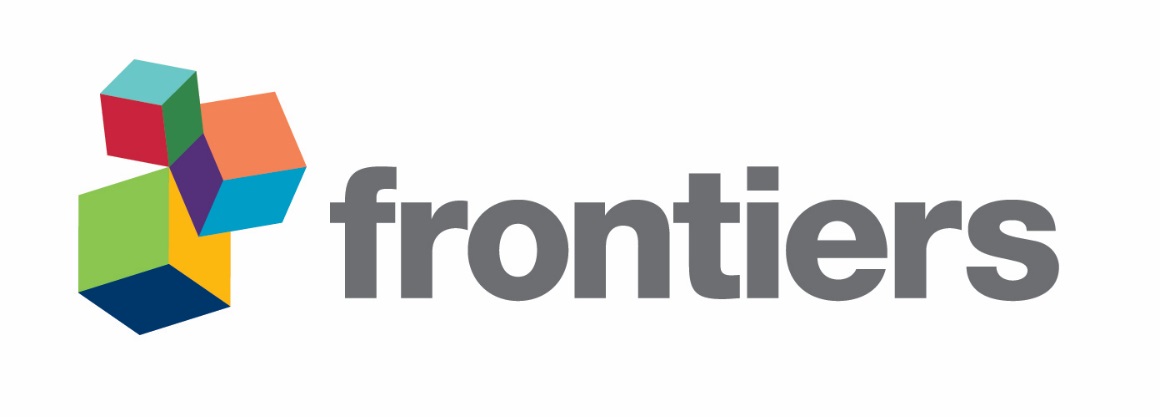
**
